# Natural Polymers for Organ 3D Bioprinting

**DOI:** 10.3390/polym10111278

**Published:** 2018-11-16

**Authors:** Fan Liu, Qiuhong Chen, Chen Liu, Qiang Ao, Xiaohong Tian, Jun Fan, Hao Tong, Xiaohong Wang

**Affiliations:** 1 Department of Tissue Engineering, Center of 3D Printing & Organ Manufacturing, School of Fundamental Sciences, China Medical University (CMU), No. 77 Puhe Road, Shenyang North New Area, Shenyang 110122, China; liufan-sky@163.com (F.L.); qhchen@cmu.edu.cnm (Q.C.); 18856152351@163.com (C.L.); aoqiang00@163.com (Q.A.); xhtian@cmu.edu.cn (X.T.); jfan@cmu.edu.cn (J.F.); tongh007@163.com (H.T.); 2 Department of Orthodontics, School of Stomatology, China Medical University, No.117 North Nanjing Street, Shenyang 110003, China; 3 Center of Organ Manufacturing, Department of Mechanical Engineering, Tsinghua University, Beijing 100084, China

**Keywords:** 3D bioprinting, natural polymers, rapid prototyping (RP), organ manufacturing, implantable bioartificial organs, regenerative medicine

## Abstract

Three-dimensional (3D) bioprinting, known as a promising technology for bioartificial organ manufacturing, has provided unprecedented versatility to manipulate cells and other biomaterials with precise control their locations in space. Over the last decade, a number of 3D bioprinting technologies have been explored. Natural polymers have played a central role in supporting the cellular and biomolecular activities before, during and after the 3D bioprinting processes. These polymers have been widely used as effective cell-loading hydrogels for homogeneous/heterogeneous tissue/organ formation, hierarchical vascular/neural/lymphatic network construction, as well as multiple biological/biochemial/physiological/biomedical/pathological functionality realization. This review aims to cover recent progress in natural polymers for bioartificial organ 3D bioprinting. It is structured as introducing the important properties of 3D printable natural polymers, successful models of 3D tissue/organ construction and typical technologies for bioartificial organ 3D bioprinting.

## 1. Introduction

An organ is a collection of multiple tissues with particular physiological functions. The human body is made of about 80 organs according to a classification principle [1]. Each of the organs performs very important physiological functions. At present, the only effective therapy for organ deformity/defect/failure is through allograft transplantation. However, the severe donor organ shortages, the long-term treatment of immunosuppressive drugs, the life-long side effects of immune complications, and the extremely high costs of donor organs, have greatly limited its clinical applications [2].

There is an increasing demand for manufacturing bioartificial organs to repair/replace/restore the damaged/deformed/failed organs. This demand is enormous for all types of organs, but especially for the visceral organs, such as the liver, heart, kidney, lung, and stomach, related to chronic and acute failures [3,4]. A typical example is that in the United States of America the treatment of organ failures involves 34 million surgical procedures per year with less than one tenth of donors [5]. Only in 2013, there were 117,040 patients in this country who needed organ transplantation with near 28,053 suitable organs available [6].

The serious shortage in organ donor supply, together with the side effects of allograft rejections and extremely high costs of donor organs with respect to allograft organ transplantation has fueled numerous strategies for organ manufacturing over the last several decades [7,8,9,10,11]. Organ manufacturing is an interdisciplinary field that needs to integrate a large scope of talents, such as biology, materials, chemistry, physics, mechanics, informatics, computers, and medicine, to design and build bioartificial organs with essential multiple cell types, hierarchical vascular/neural/lympatic networks, heterogenous extracellular matrices (ECMs), and expected biological/biochemical/physiological functions [12,13,14].

Recently, three-dimensional (3D) bioprinting, also named as rapid prototyping (RP), additive manufacturing (AM), and solid freeform manufacturing (SFM), technologies have emerged as a promise way to produce bioartificial organs through an automatic layer-by-layer deposition method [15,16,17]. The most obvious characteristic of 3D bioprinting technologies is to print living cells together with polymeric hydrogels and/or other bioactive agents as ‘bioinks’ under the instructions of computer-aided design (CAD) models. Multiple cell types can be encapsulated in different polymeric hydrogels and deposited (or delivered) simultaneously.

Polymeric hydrogels are 3D hydrophilic networks which can absorb and retain large amount of water and gel under certain biological/physical/chemical and/or biochemical/physiological/pathological conditions. The polymeric hydrogels which have been used as ‘bioinks’ for tissue/organ 3D bioprinting including both natural and synthetic polymers and their combinations [18,19,20]. The hydrogels are usually formed by physical (reversible), chemical (reversible or irreversible) or biochemical (irreversible) crosslinking of homopolymer or copolymer solutions. Compared with synthetic polymers, the natural polymeric hydrogels can provide a benign and stable environment for cells/especially stem cells to grow, migrate, proliferate, and/or differentiate inside.

Over the last decade, natural polymers as the main components of 3D printable ‘bioinks’ have played a critical role in various 3D bioprinting technologies during the layered 3D construction processes. Cell behaviors within the natural polymeric hydrogels can be controlled through changing the physical/chemical/biochemical/physiological properties of the employed polymers. With these advanced sciences and technologies Professor X. Wang has overcome all the bottleneck problems, such as large scale-up tissue/organ engineering, living tissue/organ preservation, hierarchical vascular/neural network construction, complex bioartificial organ manufacturing, partly/fully controlled stem cell engagement (or differentiation), which have bewildered tissue engineers for more than three decades. In this review, we provide a comprehensive overview of 3D bioprinted natural polymers which have been used frequently in 3D bioprinting technologies. The intrinsic/extrinsic properties of the natural polymers for bioartificial organ 3D bioprinting have been outlined. Typical successful models have been highlighted.

## 2. Properties of Natural Polymers

Natural polymers, also referred to as bio-derived materials, occur in nature and can be extracted using physical or chemical methods. Examples of naturally occurring polymers include silk, wool, deoxyribonucleic acid (DNA), cellulose and proteins. These polymers have been widely applied in many industry areas, such as foods, textiles, papers, woods, adhesives, and pharmacies. 

Some natural polymers, such as gelatin, alginate, fibrinogen, hyaluronic acid (or hyaluronan), are water-soluble, meaning that these polymers can dissolve in cell friendly inorganic solvents, such as cell culture medium and phosphate-buffered saline, to form solutions/hydrogels. The solution or hydrogel states of the natural polymers hold certain fluidity which makes them possible to be 3D printed layer-by-layer under the instructions of CAD models using the discrete-stacking RP, AM, or SFM principles [18,19,20]. Before, during, and after the 3D bioprinting process, the polymeric solutions/hydrogels offer cells and/or biomolecules (i.e., bioactive agents) a mild biomimic environment and facilitate cellular activities (i.e., cell-response bioactivities).

Theoretically, any natural polymers which have a sol-gel phase transition (i.e., gelation point) under certain conditions can be printed through an automatic layer-by-layer deposition method. In fact, very few natural polymers can be printed in layers at cell benign conditions (such as room temperature) without the help of physical/chemical/biochemcial crosslinking the incorporated polymer chains. This is due to that very few natural polymers can meet all the basic requirements for cell/tissue/organ 3D bioprinting [21,22,23].

During and after the 3D bioprinting process, natural polymers have played several essential roles in multiple cellular/biomolecular self/inter actions, homogeneous/heterogeneous histogenesis modulations/integrations/coordinations, and bioartifical organ generations/maturations. These essential roles include providing suitable accommodations for cellular/biomolecular activities (e.g., growth, migration, aggregation, proliferation, differentiation/mobilization, infiltration, coaction), enough space for extracellular matrix (ECM) patterns (e.g., formation, secretion, orientation), biophyscial/chemical cues for tissue/organ morphologies (e.g., formation, modeling, reshaping), and hierarchical vascular/neural/lymphatic network settings (e.g., construction, integration, figuration). The unexpected processing parameters, such as extreme temperatures, organic solvents and water deficiencies, which negatively influence the bioactivities of the encapsulated cells and/or biomolecules can be avoided effectively [24,25,26].

Over the last decade numerous natural polymers, such as gelatin, alginate, collagen, silk, hyaluronan, chitosan, fibrinogen, agar (or agarose), and decellularized extracellular matrix (dECM), have been printed either alone or together with other polymers as the main component of ‘bioinks’. Each natural polymer has special physical characters (in response to various external stimuli, such as temperature, light, pH, magnetism, and electricity), chemical properties, processing methods, cell-material interactions, and biomedical applications. Some other natural polymers, such as growth factors, resin, matrigel, poly (acrylic acid), polypeptide-DNA, anticoagulants (including heparin and coumarin), and polysaccharide (including dextran and starch), have been used occassionally in 3D bioprinting areas [27,28,29,30]. Small molecular materials and/or bioactive agents (or signals), such as ceramics, salts, sugars (including monose and fructose), and cryoprotectants (including dimethyl sulfoxide and glycerol), can be incorporated into the polymeric solutions or hydrogels through different approaches or protocols. These natural polymers have huge scientific research value and extravagant commercial profit in various biomedical fields. The currently available natural polymeric ‘bioinks’ as off-the-shelf products have been summarized in Table 1 [31,32,33,34,35,36,37,38,39,40,41]. 

There are three prominent characteristics of these natural polymers for 3D bioprinting: good biocompatibility, poor mechanical strength and rapid biodegradability. In the following section, several representative natural polymers for organ 3D bioprinting have been reviewed in details from the aspects of 3D printability, biocompatibility, physical/chemical/biochemical crosslinkability, biodegradability and structural stability.

## 3. Natural Polymers for Tissue/Organ 3D Bioprinting

### 3.1. Alginate

**Figure 1:**
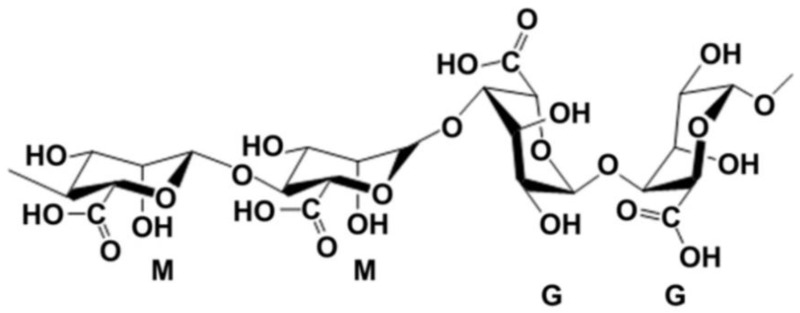
Structure units of alginate molecule [42].

Alginate, also called algin, is an anionic polysaccharide derived from brown algae. The term alginate is usually used for the salts of alginic acid, which is composed of β-d-mannuronic acid (M block) and α-l-glucuronic acid (G block) (Figure 1), and can refer to all the derivatives of alginic acid and alginic acid itself [42]. Alginate can dissolve in water and be chemically crosslinked by divalent cations, such as calcium (Ca^2+^), strontium (Sr^2+^) and barium (Ba^2+^) ions, which has been particularly attractive in wound healing, drug delivery and regenerative medicine [43,44,45]. The ratio between the M and G block is closely related to physiochemical properties of the alginate solution. A higher G/M ratio provides rigidity to polymeric structure and mechanical properties, while lower G/M ratio increases the flexibility [46,47].

Alginate and composite alginate hydrogels have been frequently used as cell-laden ‘bioinks’ in some 3D bioprinting technologies because their good biocompatibility (i.e., low toxicity, non-immunogenicity), rapid biodegradability, and chemical gelling capability (i.e., crosslinkable characteristic) [48]. Alginate related 3D bioprinting processes can be completed through different mechanisms, such as cell-laden hydrogel biopplotting in a plotting medium (crosslinker pool), coaxial nozzle-assisted crosslinking deposition with crosslinker spraying over the extruded cell-laden hydrogel, pre-crosslinked alginate hydrogel coextruded with cells [49,50]. Each of these 3D bioprinting technologies has pros and cons in tissue/organ 3D printing areas.

The first alginate application in 3D bioprinting is in 2003 by Professor X. Wang, when sodium alginate was printed with gelatin hydrogel as an additive [51]. Since the physical sol-gel transition of sodium alginate solution is below 0 °C, which is much low than that of the gelatin solution (28 °C), it is difficult for the alginate hydrogel to be printed alone at room temperatures [52,53]. Physical blending and chemical crosslinking approaches have been employed in 3D bioprinting technologies from then on.

During the 3D bioprinting processes, the viscosity of the cell-laden alginate hydrogel depends largely on the polymer concentration, molecular weight, cell phenotype and density. Typically, when cells are embedded in an alginate hydrogel with high polymer concentration, their bioactivities are greatly restricted after chemical crosslinking. Meanwhile lower concentration of alginate hydrogel facilities higher cell viability and proliferation capability. Nevertheless, when the concentration of the alginate hydrogel is reduced, the mechanical strength of the 3D construct drops sharply even after chemical crosslinking. An optimized alginate concentration is necessary for a typical 3D bioprinting technology to ensure the favorable cell viability and printing resolution. In this respect, Park et al. summarized the suitable concentration and molecular weight of alginate hydrogel for soft tissue bioprinting in consideration of mechanical property (i.e., module), printability and cell viability. It was reported that the alginate hydrogel composed of 3 wt % alginate with a mixture of low and high molecular weights in a 1:2 ratio displayed a good printability, favorable cell viability and proliferation capability after printing for seven days [54]. Additionally, the blends of alginate hydrogel with other polymers, such as gelatin, collagen and nano-fibrillated cellulose can effectively enhance the cellular activities as well as the printing resolution from 1000 μm to 400–600 μm with a 300 μm diameter nozzle [55]. Other processing parameters including nozzle size, surrounding temperature, scanning speed and dispensing pressure can also influence the printing resolution.

There are two common phenomena in alginate 3D bioprinting. The first one is that it is hard for the pure alginate solution with a low polymer concentration to be printed layer-by-layer into a high scale-up construct due to its low phase changing temperature (i.e., sol-gel transition point) and shear thinning characteristic [42]. Another common phenomenon is that the slow biodegradation velocity of alginate molecules can be tuned by oxidation procedures [56]. The oxidized alginate molecules with an appropriate degradation rate may have more potential usage in organ 3D printing. In this respect, Jia et al. focused on oxidizing alginate molecules to control the alginate printability and degradability. Accurate lattice-structures (with higher accuracies) could be obtained through optimizing the oxidized alginate solutions before printing. Human adipose-derived stem cells (hADSCs) could be loaded in the oxidized alginate solutions as well. The oxidation percentage (ox.) and concentration (conc.) of the alginate had been summarized in their research. The results indicated that 0% ox.-8% conc. alginate induced a round cell morphology that might apply to chondrogenesis, while 5% ox.-15% conc. alginate associated with an increased spreading cell phenotype that might improve osteogenesis. The 5% ox.-15% conc. alginate was recommended as the most appropriate formulation of their ‘bioinks’ for 3D bioprinting. The available literature on alginate as ‘bioinks’ for 3D bioprinting technologies is summarized in Table 2 according to the chronological sequence [57,58,59,60,61,62,63,64,65,66,67,68,69,70,71,72,73,74,75,76,77,78,79,80,81,82,83,84,85,86,87,88].

### 3.2. Gelatin

**Figure 2:**
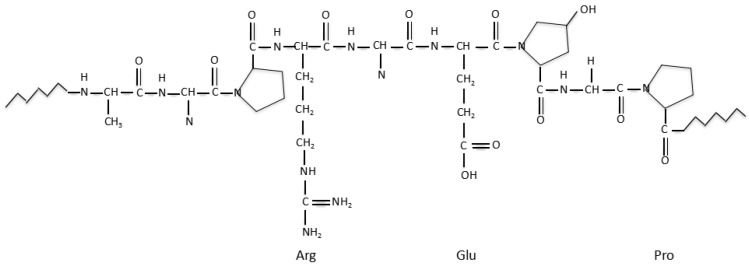
Molecular structure of gelatin.

Gelatin is a partial hydrolyzed protein by breaking the triple helix of collagen into single-strain molecules. It is a thermal-response linear polymer. Gelatin and its derivatives have been widely applied in 3D bioprinting due to their excellent biocompatibility, high water-adsorbing capacity, rapid biodegradability, non-immunogenicity and unique 3D printability (Figure 2) [53,54,55,56,57,58,59,60,61,62,63,64,65,66,67,89]. Gelatin solution has a unique sol-gel transition at 28 °C, which is corresponding to the melt point of gelatin hydrogel.

Before printing, the bulk gelatin-based polymers need to dissolve in an inorganic solvent, such as phosphate-buffered saline or cell culture medium, to form fluidic solutions with a suitable viscosity. Any types of cells and/or bioactive agents (such as growth factors, hormones, anticoagulants and cryoprotectants) can be incorporated into the gelatin solutions [90]. After the cells and/or bioactive agents are mixed with the gelatin-based solutions, the cells and/or bioactive agents are suspended in the solutions. When the solutions are cooled below 28 °C, the fluidic solutions become sticky hydrogels accompanying with a sol-gel transition phenomenon. Physical crosslinking (i.e., gelling or gelation) among the gelatin molecules happens during the sol-gel transition processes. The incorporated cells and/or bioactive agents are reversibly embedded (or encapsulated) by the polymeric chains. Other natural polymers, such as alginate, chitosan, fibrinogen, hyaluronan, collagen, agar and matrigel, can be incorporated into the gelatin solutions as additives, leading to the gelatin solutions/hydrogels being printed alone or together with the other polymeric additives as the cell/bioactive agent-laden ‘bioinks’ [91]. Chemical crosslinking of the polymeric molecules is an effective approach to enhance the mechanical properties and structural stability of the 3D printed constructs. Ten years later, these techniques have been world widely adapted, duplicated or copied by many other groups, such as the A. Atala’s at Wake Forest School of Medicine in North Carolina [92], and the J.A. Lewis’s in Harvard University [93].

Thus, the special thermal response character of the gelatin based solutions/hydrodgels allow cells and/or bioactive agents to be injected or extruded through the nozzles/needles of 3D bioprinters and subsequently piled up in layers at a relatively benign environmental temperature between 1–28 °C. During and after the 3D printing processes, the gelatin based solutions/hydrogels act both as the backbones (such as the ECMs) to support the structural integrity of the 3D constructs and the accommodations for cells and/or bioactive agents within the predefined 3D constructs.

Especially, in extrusion-based 3D bioprinting technologies, the printing processes can be tuned to be no harm (or adverse effect) to the incorporated cells and/or bioactive agents with respect to the applied parameters, such as shear forces, squeeze rates and nozzle sizes. Cell viability, especially stem cell proliferation and differentiation capabilities, can be preserved. Extremely high cell viabilities (i.e., 100% percentage) can be achieved by optimizing the printing parameters, such as the nozzle diameter, acting force, printing speed and surrounding temperature [11,12,13,57,58,59,60,61,62]. Additionally, the presence of arginine-glycine-aspartic acid (or Arg-Gly-Asp, RGD) peptide domains in the gelatin molecules may have favorable effects on cell activities, such as migration and differentiation [94].

Obviously, there are two distinguished disadvantages of the gelatin-based hydrogels in organ 3D printing areas: one is the low mechanical strength and the other is the structural instability under physiological conditions (such as 37 °C). It is observed that when the printed cell-laden 3D constructs are put into culture medium at about 37 °C, the physical crosslinked gel states (or structures) break down quickly. This is because that above the melt point of 28 °C, the physical crosslinking bonds in the gelatin molecules disorganize, leading to the collapses of the structural integrity of the 3D constructs. In another word, the gelled gelatin-based constructs disperse immediately in the culture medium due to the reversible physical crosslinking bonds. The 3D printed gelatin-based constructs need to be further strengthened to yield a stable structure.

Consequently, various physical blending and chemical crosslinking techniques have been applied to improve the mechanical property and structural stability of the 3D printed constructs. For example, gelatin molecules have been chemically modified with methacrylamide groups to yield a natural/synthetic hybrid hydrogel (i.e., gelatin/methacrylate, GelMA). The hybrid GelMA hydrogel can be photopolymerized in the presence of a water-soluble photoinitiator and ultraviolet (UV) [95]. It is reported that the increase of the gelatin or GelMA proportion (or concentration) can significantly elevate the viscosity and printability of the composite GelMA hydrogel. The UV crosslinking of GelMA can dramatically enhance the mechanical properties and shape fidelity of the 3D printed constructs. The shape fidelity can be subsequently improved from 1100–1300 μm to 350–450 μm using a 200 μm diameter nozzle. Cell survival capability in the GelMA hydrogel can reach 83% with a cell density of 1.5 × 10^6^ cells/mL, 10% GelMA hydrogel and 60 s UV exposure. HepG2 in the GelMA constructs can retain a high cell viability for at least eight days, possibly due to the protective effect of the composite GelMA hydrogels, in which the shear stress applied on the encapsulated cells resulting from the friction of the hybrid cell-laden hydrogels with the nozzle walls.

Another example is that the gelatin-based hydrogels can be chemically crosslinked by some small bioactive agents (or chemicals), such as glutaraldehyde and CaCl_2_ [96]. The rapid biodegradation property of the gelatin-based hydrogels, such as the gelatin, gelatin/alginate, gelatin/chitosan, and gelatin/fibrinogen, has been greatly enhanced through the chemical crosslinked polymer chains. Typical chemical crosslinked gelatin based hydrogels contain the glutaraldehyde crosslinked gelatin molecules, CaCl_2_ crosslinked alginate molecules, tripolyphosphate (TPP) crosslinked chitosan molecules, and thrombin polymerized fibrinogen molecules. Much more stable 3D constructs can be obtained through double/triple crosslinking the composite (or hybird) gelatin-based hydrogels. For instance, gelatin/algiante/fibrinogen hydrogel is a natura/natural/natural hybrid hydrogel, it can be effectively stabilized through double crosslinking the polymer molecules in the hybrid hydrogel using both CaCl_2_ and thrombin solutions (i.e., using CaCl_2_ to crosslink the algiante molecules and thrombin to polymerize the fibrinogen molecules in the gelatin/alginate/fibrinogen hydrogels). Though progressive loss of the uncrosslinked gelatin molecules in the long-term in vitro cultured gelatin/alginate/fibrin constructs has been detected, the living 3D constructs can be maintained properly over four weeks until the new tissue/organ generation [11,12,13]. The representative literature on the gelatin-based hydrogels as ‘bioinks’ in tissue/organ 3D bioprinting is summarized in Table 3 except those appeared in Table 2 [97,98,99,100,101,102,103,104,105,106].

### 3.3. Hyaluronic Acid

**Figure 3:**
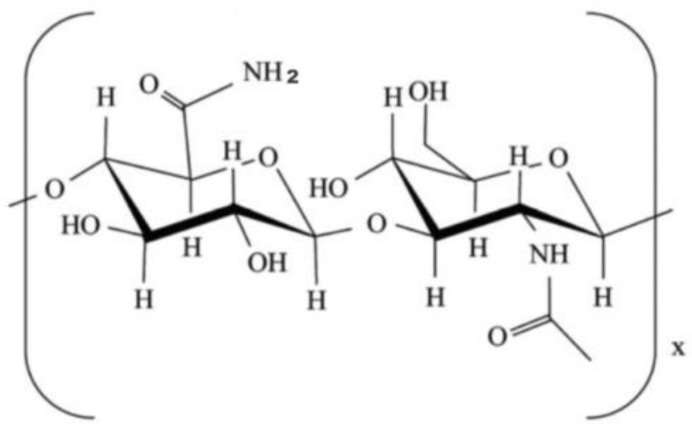
Structure unit of hyaluronic acid (HA).

Hyaluronic acid (HA) or hyaluronan is a polysaccharide existing in living organisms composed of d-glucuronic acid and N-acetyl-d-glucosamine (Figure 3) [107]. As a component of ECM, HA has excellent biocompatibility and biodegradability, which has played an essential role in cell proliferation, angiogenesis and cell-receptor interactions. HA can be rapidly degraded (i.e., glycolytically degraded in a glycolytic pathway) by hyaluronidase, β-glucuronidase and β-N-acetyl-glucosaminidase into low molecule weight hyaluronic acid and oligosaccharides [108,109]. It is a lubricating hydrophilic polymer that can form highly viscous hydrogels at low concentrations, and can be applied as an additive to alter the viscosity of the above mentioned gelatin based ‘bioinks’.

Like most of the natural polymers, HA has poor mechanical properties which result in low shape fidelity during 3D bioprinting. Numerous modifications have been carried out to improve the mechanical properties and shape fidelity of the HA based 3D printing processes. These modifications include physical or chemical crosslinking of HA with other polymers. For example, hyaluronic acid methacrylate (HAMA) is a natural/synthetic hybrid polymer generated by photochemically crosslinking HA and methacrylate using UA-light source [110]. Emphasis should be given that although HAMA has altered the poor physical properties of the HA, the bioinert characters of the HAMA, including non-biodegradability of polymethacrylate (i.e., PMA), high hardness (or stiffness) and low shape fidelity, have greatly limited its application in organ 3D bioprinting areas [111].

Another example is the gelatin or GelMA blended HA (e.g., HAMA-GelMA). The printing parameters of the hybrid HAMA-GelMA (i.e., concentration or composition ratio) directly affect the mechanical properties of the printed 3D constructs, the degradation rate of the natural components in the 3D constructs, as well as the cell spreading, adhesion and proliferation features in the 3D constructs. In this respect, Camci-Unal et al. reported that the addition of 1% *v*/*w* HAMA into the GelMA hydrogel could decrease the mass swelling ratio and degradation rate, but increased the compressive moduli of the 3D constructs. No cell spreading was found within the 3D printed HAMA construct. Cell spreading could only be observed in the samples with 3% GelMA and 1% HAMA, possibly due to the existence of the cell adhesive motifs in the gelatin molecules [112].

Until the present, the hybrid HAMA-GelMA ‘bioinks’ with proper proportion of HAMA and GelMA have been applied in some tissue engineering applications, including neural, cardiovascular, cartilage and bone tissues. One example is that Duan et al. have improved the spreading and adhesive capabilities of the human aortic valvular interstitial cells, and the phenotype maintaining ability of the fibroblasts in the 3D printed trileaflet valve conduits through increasing the GelMA concertration in the hybrid HAMA-GelMA hydrogels (with decreased stiffness) [113]. Similar results have been found by Skardal and coworkers through changing the ratio of HAMA and GelMA, Skardal et al. reported that a high ratio of HAMA/GelMA would result in a stiffer construct, but poor cell adhesive ability, while low ratios lead to poor mechanical strengths but better cell adhesions. The 80/20 ratio of HAMA/GelMA was an optimal choice with all aspects considered [101].

### 3.4. Collagen

**Figure 4:**
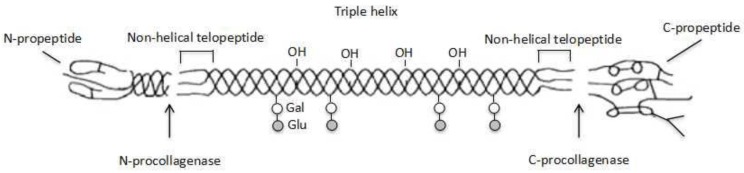
Schematic diagram of collagen molecule.

Natural collagen has been widely used as a scaffold material for tissue engineering over the last several decades (Figure 4). It can significantly improve the adhesion, proliferation and differentiation capabilities of osteoblasts, chondroblasts, and mesenchymal stem cells on the porous scaffolds [114]. Especially, the pore size of 50–150 μm of the collagen scaffold facilitates cell seeding on the surface of the pores. It is supposed that the collagen molecule, which has the same RGD peptide domains with gelatin, can be recognized by integrin receptors on the cell membrane and promote cell adhesion and proliferation. Nonetheless, the properties of acid-soluble collagen solution can be easily affected by the potential of hydrogel (pH) and temperature, which make it difficult for the collagen solution to be 3D printed at ambient conditions. The reason is that collagen molecules tend to assemble to form hydrogel and can be rapidly degraded by collagenases and metalloproteinases into amino acids when the solution is neutralized at 37 °C. 

Especially, collagen type I and II have been frequently employed for cartilage and bone repair scaffold 3D printing. There are three obvious advantages for the 3D printed scaffolds to be used for tissue repair. Firstly, most of the 3D printed scaffolds have scale-up go-through channels, which are different from the traditional tissue engineering porous scaffolds, and helpful for nutrient, oxygen, and metabolite transportation. Secondly, the structural morphology and material composite of 3D printed scaffolds can be gradient, which are benefit for multiple functionality realization. Thirdly, living cells can be directly incorporated into the biocompatible materials for hard/soft tissue/organ engineering. For example, the articular cartilage has a zonal ECM distribution with gradient cell density from articular surface to calcified cartilage. One of the main defects of conventional tissue engineered articular cartilage is the difficult to mimic zonal mechanical and biological properties of natural cartilage, while bioprinting technologies have the capability to fabricate well-designed constructs with accuracy cell distribution [115]. A study by Ren et al. focused on the engineered zonal cartilage by bioprinting collagen type II hydrogel constructs with a gradient chondrocyte density. In this study, collagen type II had the ability to maintain chondrocyte phenotype and played an essential role to promote chondrogenic differentiation. The 3D printed zonal cartilage had a gradient ECM distribution, which was positively correlated to chondrocyte density. Both the both the cell density and cell distribution pattern in the bioprinting process had been modulated in different zonal areas for better biological effects [116]. 

Until now, the low viscosity and fast degrading rate of the pure collagen hydrogels have seriously limited their applications as ‘bioinks’ in tissue/organ 3D bioprinting. Blending with other polymers, such as alginate, fibrin, agarose, and hyaluronic acid, has become the most common strategies to alter the viscosity, degradation rate, and printability of the natural collagen [114]. However, the blending of collagen with alginate or agarose has greatly comprised the cell viability. This is because that the ultimate biocompatibility of the blend is determined by the worst of component of the composite, such as alginate in the collagen/alginate hydrogel and agarose in the collagen/agarose hydrogel. On the contrary, a hybrid of chondrocyte-encapsulated hyaluronic acid and osteoblast-encapsulated collagen type I hydrogel was reported for osteochondral bioprinting with good results [117]. Recently, a 3D bioprinted collagen/heparin sulfate scaffold was reported to promote neurological function recovery by providing continuous guidance channels for axons and enough mechanical strength for the injured spinal cord. The heparin modification has resulted in enhanced compress modulus and binding affinity of the new secreted ECM proteins [118].

In our group, collagen I has been printed with polyurethane simultaneously using our home-made double-nozzle low-temperature 3D bioprinter for a double layer nerve repair conduit manufacture [119,120,121]. Swann cell compatibility in the double layer nerve repair conduit has been significantly improved. Meanwhile nerve repair speed inside double layer nerve repair conduit has been the has been extremely accelerated.

### 3.5. Fibrin

**Figure 5:**
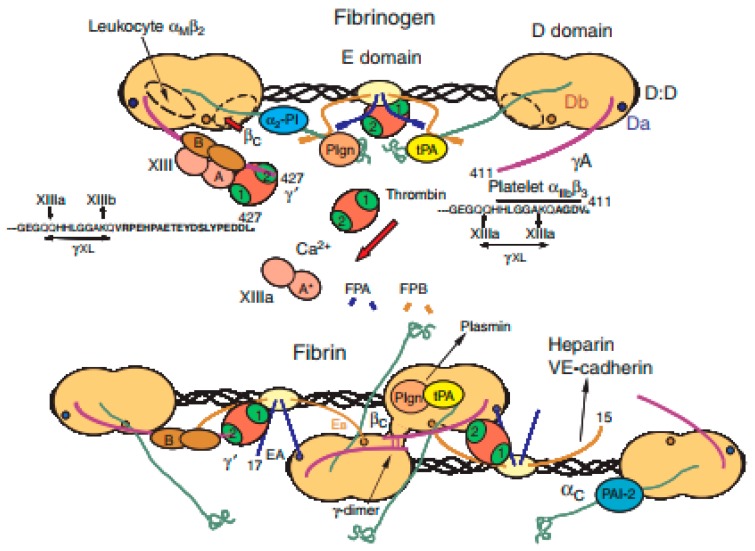
Schematic structures of fibrinogen and fibrin [121].

Fibrin is a blood-derived fibrous (i.e., non-globular) protein formed by polymerizing fibrinogen in the presence of the protease thrombin (Figure 5) [121]. Over the last several decades, fibrin has been widely used in many biomedical fields, such as pharmacy, wound healing, and tissue repair. Compared with plant-derived natural polymers, such as alginate and agarose, fibrin has excellent biocompatibilities in biomedical fields. In the context of cell viability, fibrin hydrogel, has super cytocompatibility for cell encapsulation, delivery and culture [122,123,124]. With respect to the degradation velocity, fibrin can degrade rapidly due to the presence of proteolytic enzymes [125].

Despite the superior biological/biochemical/biomedical properties, the low viscosity, rapid gelation process, quick degradation velocity and limited mechanical strength of the fibrin-based hydrogels all need to be addressed for organ 3D bioprinting with anti-suture capability. Especially, when the fibrinogen solution was printed alone with cells, the rapid gelation process is difficult to control to form stable 3D constructs. An effective solution is to blend the fibrinogen solution with other chemical crosslinkable natural polymers, such as gelatin, alginate, hyaluronan, and collagen. 

Like alginate, the first report of fibrin in 3D bioprinting is in 2007 by Professor X. Wang, in which fibrinogen was acted as an additive of the gelatin based hydrogel for hepatic tissue or vascularized hepatic tissue manufacturing [21]. The physical blending and chemical crosslinking fibrinogen with gelatin molecules can evidently avoid the collapse of the 3D printed constructs, slow down the polymer degradation rate and amend the structural stability. Nowadays, the physical blending and chemical crosslinking have rapidly expanded to much more complicated hybrid polymeric ‘bioinks’, such as the gelatin/fibrinogen/alginate, gelatin/fibrinogen/chitosan gelatin/fibrinogen/hyaluronan, and gelatin/fibrinogen/hyaluronan/glycerol (dimethyl sulfoxide, dextran-40, or heparin) [54,55,61,62]. The combination of physical blending and chemical crosslinking is a successful way to make the natural polymeric ‘bioinks’ to fulfill various requirements for tissue/organ 3D bioprinting, such as providing adequate benign environments for cell encapsulation during and after the printing processes.

Among all the fibrin containing ‘bioinks’, gelatin/alginate/fibrin has been chosen as an optimal constituent for complicated organ 3D bioprinting [54,55,61,62]. Partly due to that the composite gelatin/alginate/fibrin hydrogel can be double reinforced by polymerizing the fibrinogen molecules using thrombin and crosslinking the alginate molecules using CaCl_2_. After the double chemical crosslinking, the gelatin/alginate/fibrin hydrogel has exceptional mechanical properties, excellent cytocompatibilities, and extraordinary physiological functions. Correspondingly, the compressive and elastic modulus of the 3D bioprinted constructs have been strengthened remarkably [126]. 

An outstanding character of the gelatin/alginate/fibrin hydrogel is that all the cells and bioactive agents can be incorporated and 3D bioprinted without reducing their bioactivities. Stem cells, such as adipose-derived stem cells (ADSCs), can be induced into various target tissues/organs with proper growth factor engagement [54,55,61,62]. It is regarded as a milestone in large vascularized organ manufacturing fields. Until now, stem cells have been regarded as the ideal cell types for large scale-up vascularized organ 3D bioprinting [127,128,129].

Recently, fibrin has been used in some other 3D bioprinting technologies, such as skin and adipose organ engineering, with excellent cell and tissue compatibilities [106,130]. For example, Hakam et al. reported the gelatin/fibrin hydrogel for skin bioprinting. The hybrid hydrogel with 1:1 *v*/*v* gelatin/fibrin ratio was selected as an optimal composition to evaluate the water/glucose absorption capability, polymer degradation rate, mechanical compression situation and water vapor transmission. As a result, this hybrid hydrogel could provide the cell pellets within 200–250 μm in diameter, with better cell viability [105]. Additionally, in situ printing of fibrin-collagen hydrogels with amniotic fluid-derived stem cells could result in increased wound closure rates, as well as increased vascularization of the regenerating tissues [131].

### 3.6. Chitosan

**Figure 6:**
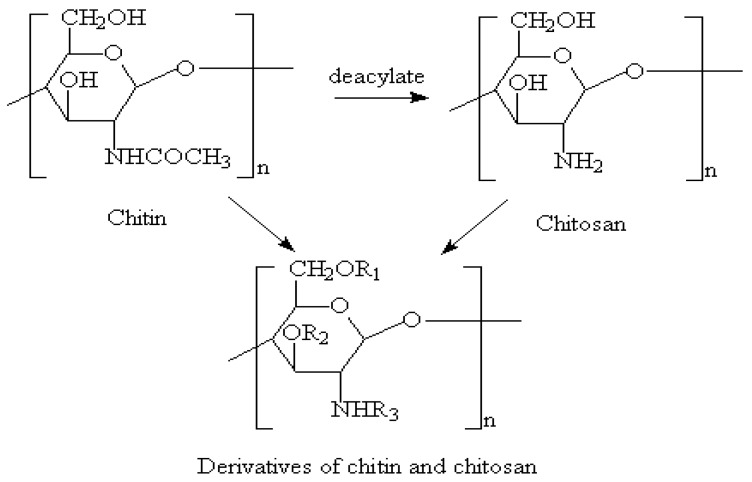
Molecular structure of chitosan [122].

Chitosan is a natural polysaccharide (derived from shrimp shell) formed by deacetylation of chitin, which has been applied in many biomedical fields, such as bone, skin and cartilage repair, due to its low or non-toxic, antibiotic and biodegradable properties (Figure 6) [132,133,134,135,136,137,138]. Chitosan can be biodegraded by lysozymes into amino-sugars [119,139]. Similar to alginate and hyaluronan, the poor mechanical strengths and slow gelation properties of the chitosan solutions have obvious hindered its application in organ 3D printing areas.

Similar stabilization strategies have also been used in chitosan 3D bioprinting by Professor X. Wang [97]. The mechanical properties can be intensified as expected by physical blending and chemical crosslinking chitosan with other supportive polymers, such as alginate, gelatin and collagen [97]. As a normal approach, high viscosity of chitosan is recommended in the extrusion-based hybrid polymeric hydrogel 3D bioprinting technologies. Recently, collagen/chitosan, alginate/chitosan, gelatin/alginate/chitosan have been frequently utilized as ‘bioinks’ in various organ 3D bioprinting areas. Exceptionally, chitosan itself can be chemically modified to improve its printability at suitable pH (7–7.4) with no detriments on its biocompatibility and biodegradability.

### 3.7. Agarose

**Figure 7:**
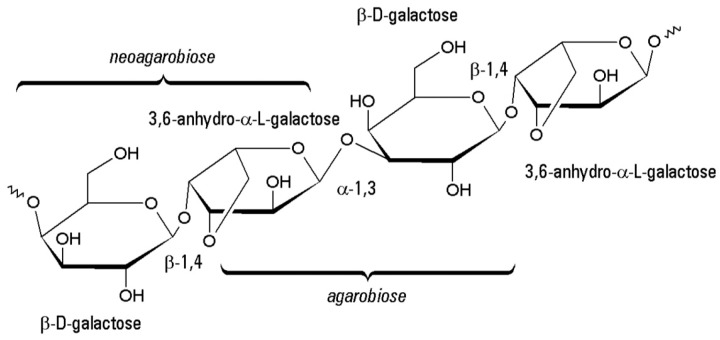
Molecular structure of agarose.

Agarose derived from the cell wall of red algae is a naturally linear polysaccharide that mainly composes of β-d-galactopyranose and 3,6-anhydro-α-l-galactopyranose (Figure 7) [140]. It is another thermal-response natural polymer besides gelatin with a liquefaction temperature approximate 30 °C, which is suitable for the extrusion-based 3D bioprinting processes [141].

Unlike gelatin, the gelling temperature of agarose depends on the polymer concentration. Agarose has no cell adhesion motifs and the cell encapsulation capacity is poor. It is usually used as bacterium culture substrates. Recently, it appears as a modified polymer in 3D bioprinting by blending or crosslinking with other supportive components in a hybrid polymeric hydrogel [142]. For example, an optimal composite hydrogel with 50% *v*/*v* Matrigel and 3% *w*/*v* agarose can support the growth and adhesion of intestinal epithelial cells, especially at a constant temperature of 37 °C [143].

### 3.8. Decellularized Extracellular Matrix (dECM)

Decellularized extracellular matrix (dECM) is a mixture of natural polymers, which is obtained from decellularization of different animal tissues, such as skin, small intestinal submucosa, and liver [144]. After decellularization, the composition and topology of the original tissues can be highly remained, which can provide tissue-specific microenvironments for preserving cell-specific functions. The decellularization processes can be either physical, chemical, biochemical (e.g., enzymatic) or their combinations, which can affect the final dECM compositions [145]. The resulted dECM-based solutions gel immediately beyond 15 °C and form physically crosslinked hydrogels. It was reported that, procine-liver-derived dECM could be used as a functional substrate for hepatocyte culture. The liver-specific dECM could maintain hepatocyte functions through albmin secretion, mRNA expression of bilesalt export pump (BSEP) and sodium taurocholate co-transporting polypeptide (NTCP), which was regarded as a promising scaffold material in tissue 3D bioprinting [146].

For organ 3D bioprinting, dECM can provide cells with customized milieus. Due to its low viscosity, dECM ‘bioinks’ often need other supportive polymers to provide basic 3D printability and shape fidelity. For example, an adipose-derived dECM/polycaprolactone (PCL) hydrogel with encapsulated ADSCs has been printed by a multi-head tissue building system and resulted in a high cell viability (>90%) [147].

Currently, there is a growing interest on using dECM-derived ‘bioinks’ for a variety of customized bioartificial organ 3D bioprinting, both in academic and industrial settings. Some dECM-derived ‘bioinks’ have been examined as potential replacements for clinical applications. Nervetheless, the clinical success depends largely on how well the mechanical property preserved. Although dECM has remarkable advantages for tissue/organ-specific function preservation, it still faces many other challenges for complex organ 3D bioprinting. Firstly, it is difficult to efficiently remove antigenic epitopes to eliminate immune responses created by the allogeneic or xenogeneic recipients of dECMs. Secondly, residual DNA or nuclear materials are retained more or less in the dECMs, which probably affects the encapsulated cell behaviors. Lastly, the extremely weak mechanical properties, poor construction resolution, remarkable shape shrinkage and rapid degradation rate are major problems to be solved in the future [148].

## 4. Typical Organ 3D Bioprinting Technologies

Unlike tissues, which can be printed using simple 3D printers and ‘bioinks’. All the organs have large scale-up heterogeneous cell/tissue components with complex vascular, neural or lymphatic networks. The complexity of the geometrical architecture and material constituent determines the difficulty levels of the organ 3D bioprinting technologies.

Generally, natural polymers as the main component of various ‘bioinks’ should meet several basic requirements for a successful organ 3D bioprinting as well as clinical applications: (1) biocompatible (i.e., nontoxic or no obvious toxicity); (2) biodegradable (vs nonbiodegradable polymers can be used as supportive structures); (3) biostable with strong enough mechanical strength; (4) bioprintable (processable); (5) biostorable in a proper period.

**Figure 8:**
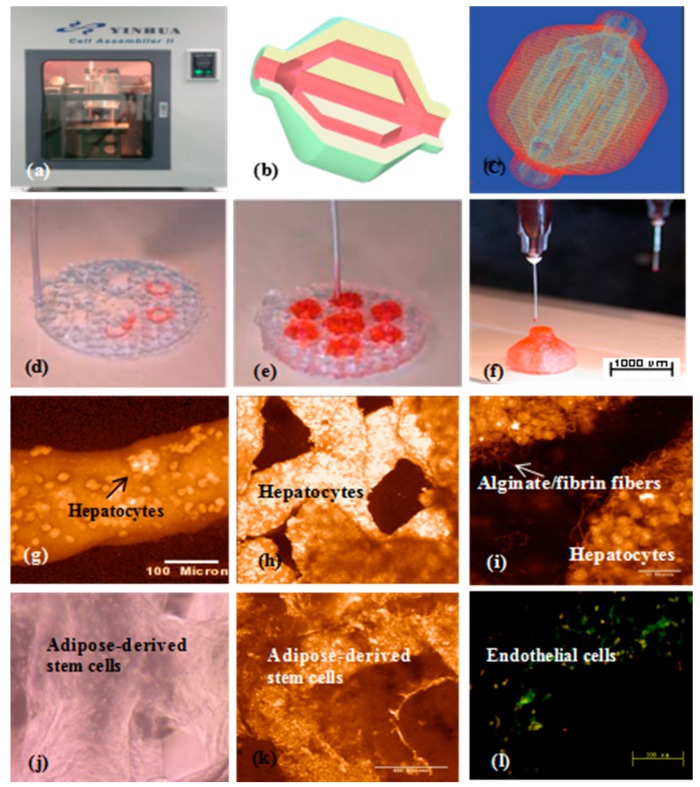
A large scale-up 3D printed vascularized organ (i.e., adipose tissue) constructed through the double-nozzle (syringe) 3D bioprinter: (**a**) The 3D printer; (**b**) a computer-aided design (CAD) model containing a branched vascular network; (**c**) a CAD model containing the branched vascular network; (**d**) 3D bioprinting with ADSCs encapsulated in the gelatin/alginate/fibrin hydrogel and hepatocytes encapsulated in the gelatin/alginate/chitosan hydrogel before epidermal growth factor (EGF) engagement, immunostaining with pyrindine (PI) for cell nuclei in red; (**e**) several 3D printed layers of the construct; (**f**) half an ellipse of the 3D construct; (**g**) hepatocytes in the gelatin-based hydrogel after 3D bioprinting; (**h**) hepatocytes in a 3D printed fiber; (**i**) hepatocytes in a grid structure; (**j**) hepatocytes in a magnificant image, the crosslinked alginate/fibrin fibers can be observed; (**k**) ADSCs in the gelatin-based hydrogel after 3D bioprinting before growth factor engagement; (**l**) ADSCs in the gelatin-based hydrogel after 3D bioprinting after EGF engagement, CD31 immunofluorescence staining endothelial cells on day 10 after EGF engagement. Most cells located on the walls of the go-through channels were CD31 positive cells with bright color (i.e., mature endothelial cells).

In 2008, a double-nozzle extrusion-based 3D bioprinter was innovated at the center of organ manufacturing in Tsinghua University, professor Wang’s laboratory (Figure 8) [88,89]. Using this technology, two cell types with large population of cells have been simultaneously printed into large scale-up living organs (Figure 8b–h). With the updated hard- and software, both the hierarchical branched vascular templates and grid go-through (i.e., interconnected) channels have been properly integrated into the living organs under the instructions of the CAD models [149,150,151]. Much more complex structures can be accomplished through imitating those of the natural organs [152,153,154,155].

These results have certified that by proper polymeric ‘bioink’ and 3D bioprinter design, the gelatin-based hydrogels in the solid 3D constructs can serve as optimal 3D substrates that engender nutrient and growth factor infiltration, multi-cellular communication (such as endothelization or vascularization), and new organ generation (i.e., a special program that is relevant to tissue colonization and organ morphologies). As those in the single-nozzle/syringe 3D bioprinting, ADSCs entrapped in the gelatin-based hydrogels can be engaged into heterogeneous tissues for large vascular organ manufacturing. Primary hepatocytes can form functional parenchymal tissues beside the hierarchical vascular and/or neural networks in the 3D constructs. This integration of natural polymers with multi-nozzle extrusion-based 3D printing technologies has reshaped the healthcare landscapes, and will unavoidably change the lives of countless individuals and bring huge benefit to the whole human beings [156,157].

## 5. Conclusions

Organ manufacturing is an interdisciplinary field that needs to integrate a large scope of talents, such as biological, material, chemical, physical, mechanical, medical and clinical. The emerging of 3D bioprinting technologies is the integration results of mechanics with biomaterials and other sciences and technologies, such as biology, chemistry, physics, informatics, computer and medicine. Natural polymers, such as gelatin, alginate, hyaluronic acid, fibrinogen, and their combinations, have played several critical roles in various tissue/organ 3D bioprinting technologies with profound influence in cellular/biomolecular selfaction/interaction, histogenesis formation/modulation and organ construction/maturation. These polymers acted as the main components of variety ‘bioinks’ are essential for bioartificial organ 3D bioprinting to provide cell-, tissue- and organ-specific structures and functions. However, the notorious poor mechanical properties of the natural polymeric ‘bioinks’ have greatly limited their usage in complex organ 3D printing with anti-suture structures, such as the hierarchical vascular, neural and lymphatic networks. Physical blending, chemical crosslinking and the combination of both natural and synthetic polymers are effective ways to solve the geometrical, mechanical, structural, physiological and clinical problems. The better understanding of the physical, chemical and biological characteristics of the natural polymers helps to build much more complex tissues and organs with much more specific structures and functions, such as the glomeruli in the kidney and biliary networks (or biliary ducts) in the liver. This is particularly important for future individual or customized organ reverse engineering/manufacturing with predefined essential architectures, diversified material compositions, specialized cellular/biomolecular activities and expected physiological/biomedical functionalities.

## Figures and Tables

**Table 1:** Commercially available natural polymeric ‘bioinks’.

3D Bioprinting Technique	‘Bioink’ Formulation	Crosslinking Method	Bioprinter	Ref.
One/two nozzle extrusion-based 3D bioprinting	Gelatin/alginate, gelatin/chitosn, gelatin/fibrinogen, gelatin/hyluronan, gelatin/alginate/fibrinogen hydrogels	CaCl_2_/thrombin/sodium tripolyphosphate (TPP)/glutaraldehyde solutions	Yinhua Cell Assembler II	[31]
	Alginate/chitosan hydrogel	CaCl_2_ solution	EFD^®^ Nordson printer	[32]
	Nanocellulose-alginate	CaCl_2_ solution	3D discovery printer	[33]
Extrusion-based scaffold-free bioprinting	Agarose hydrogel, Novogel	Sol-gel physical transition	Novogen Bioprinter	[34]
	Polyethylene glycol (PEG)/gelatin-PEG/fibrinogen	1-Ethyl-3-(3-dimethylaminopropyl)carbodiimide (EDC) and N-hydroxysuccinimide(NHS) solutions for gelatin scaffold, thrombin solutions for fibrinogen-containing samples post-printing	EnvisionTEC 3D-Bioplotter	[35]
Inkjet-based 3D bioprinting	Alginate solution	CaCl_2_ solution after printing	MicroFab MJ-ABL piezoelectric inkjet printhead printer	[36]
	Collagen/gelatin solution	Sol-gel physical transition	Valve-based inkjet printer	[37]
	Fibrinogen solution	Thrombin solution	Custom-built printer	[38]
Fab^@^HomeTM (one/two-syringe extrusion-based 3D printing)	Gelatin/glucose-alginate hydrogel	CaCl_2_ solution after printing	Fab^@^Home Model1-3	[39]
3D-Bioplot ter^TM^ system	Alginate-PCL	CaCl_2_ aerosol + CaCl_2_ solution	Cartilage template	[40]
Laser-based bioprinting	Alginate solution	CaCl_2_ solution	ExciStarexcimer laser	[41]

**Table 2:** Alginate containing ‘bioinks’ for different 3D bioprinting applications.

3D Bioprinting Technique	‘Bioink’ Formulation	Crosslinking Method	Application	Ref.
One nozzle extrusion-based 3D bioprinting	Hepatocytes and chondrocytes in gelatin/alginate hydrogel	10% CaCl_2_ solution for 2 min after printing	Bioartificial liver or cartilage manufacturing	[51]
	Adipose-derived stem cells (ADSCs) in gelatin/alginate hydrogel	5% CaCl_2_ solution after printing	Vascular networks	[52]
	ADSCs in alginate capsules and gelatin/alginate hydrogel	5% CaCl_2_ solution after printing	Vascular networks	[53,54,55,56]
One nozzle extrusion-based 3D low-temperature bioprinting	Adipose-derived stem cells (ADSCs) in gelatin/alginate/fibrinogen/dimethylsulfoxide (DMSO) hydrogel	Double crosslinking with CaCl_2_ and thrombin after printing	No specific	[57]
	Adipose-derived stem cells (ADSCs) in gelatin/alginate/DMSO and/or dextrain-40 hydrogel	5% CaCl_2_ solution after printing	No specific	[58]
	Adipose-derived stem cells (ADSCs) in gelatin/alginate/glycerol and/or dextrain-40 hydrogel	5% CaCl_2_ solution after printing	No specific	[59]
Two nozzle extrusion-based 3D bioprinting	Adipose-derived stem cells (ADSCs) in gelatin/alginate/fibrinogen hydrogel & hepatocytes in gelatin/alginate/chitosan hydrogel	Double crosslinking with CaCl_2_ and thrombin after printing	Vascularized liver tissue manufacturing	[60]
	Adipose-derived stem cells (ADSCs) in gelatin/alginate/fibrinogen hydrogel	Double crosslinking with CaCl_2_ and thrombin after printing	Vascularized adiose tissue manufacturing	[61]
	Adipose-derived stem cells (ADSCs) in gelatin/alginate/fibrinogen hydrogel	Double crosslinking with CaCl_2_ and thrombin after printing	Bioartificial pancreas manufacturing	[62]
Mutihead deposition system (extrusion-based)	Osteoblasts & chondrocytes in polycaprolactone (PCL)/alginate solution	CaCl_2_ solution after printing	Osteochondral tissue	[63]
One nozzle extrusion-based 3D bioprinting	Cartilage progenitor cell (CPCs) in alginate solution	CaCl_2_ solution after printing	Vessel-like structure	[64]
Fab@HomeTM (one-syringe extrusion-based 3D printing)	Aortic valve leaflet interstitial cells (VICs), smooth muscle cells (SMCs) or chondrocytes in gelatin/alginate solution	300 mM CaCl_2_ crosslinking for 10 min after printing	Myocardial tissue, muscle tissue and cartilage engineering	[65]
One-nozzle extrusion-based 3D bioprinting	Myoblasts in gelatin/alginate hydrogel	CaCl_2_ solution after printing	Muscle engineering	[66]
Two-nozzle low-temperature extrusion-based 3D bioprinting	PU-ADSCs in gelatin/alginate/fibrinogen hydrogel	Double crosslinking with CaCl_2_ and thrombin	Complex organ manufacturing	[67,68]
Combined four-nozzle 3D bioprinting	Poly(lactic acid-co-glycolic acid) (PLGA)-ADSCs in gelatin/alginate/fibrinogen hydrogel-hepatocytes in gelatin/chitosan hydrogel-Schwann cells in gelatin/hyaluronate hydrogel	Double crosslinking with CaCl_2_ and thrombin	Vascularized liver manufacturing	[69]
One nozzle extrusion-based 3D bioprinting	Gelatin/alginate hydrogel	CaCl_2_ crosslinking after printing	No specific	[70]
One nozzle extrusion-based 3D bioprinting	Human adipose stem cells (hASCs) in oxidized alginate solution	CaCl_2_ crosslinking after printing	No specific	[71]
Micro imprinting	Mesenchymal stem cells (MSCs) in gelatin/alginate/hydroxyapatite (HA) mixture	2% *w*/*v* CaCl_2_ crosslinking for 10 min after printing	Cartilage tissue	[72]
One nozzle extrusion-based 3D bioprinting	Preosteoblasts and hASCs in alginate solution	1.2 wt % of CaCl_2_ flow	Hepatogenic differentiation	[73]
Mutihead deposition system (extrusion-based)	Chondrocytes in PCL/alginate solution	100 mM CaCl_2_ and 145 mM NaCl solution for 10 min	Cartilage	[74]
One nozzle extrusion-based 3D bioprinting	Human umbilical vein endothelial cells in methacrylated gelatin (GelMA)/alginate hydrogel	Photopolymerization and CaCl_2_ solution	Heart tissue	[75]
Two-nozzle extrusion-based 3D printing	Gelatin/alginate/fibrinogen/HepG_2_; gelatin/alginate/fibrinogen/hepatocyte or gelatin/alginate/fibrinogen/hepatocyte/ADSC	Double crosslinking with CaCl_2_ and thrombin solutions	Liver tumor model establishment and anti-cancer drug screening	[76,77,78]
3D-Bioplot ter^TM^ system	Alginate-PCL	170 mM CaCl_2_ aerosol + 100 mM CaCl_2_ solution	Cartilage template	[79]
Multi-head bioprinting	RGD-γ alginate/poly(-ethylene glycol)-tetra-acrylate (PEGTA)/GelMA/PCL	UV light for 30 min	Cartilage engineering	[80]
A multilayered coaxial extrusion system	A specially designed cell-responsive bioink consisting of GelMA, alginate, and 4-arm poly(-ethylene glycol)-tetra-acrylate (PEGTA)	First ionically crosslinked by calcium ions (Ca^2+^ ion) followed by covalent photocrosslinking of GelMA and PEGTA	Perfusable vasculature	[81]
One nozzle extrusion-based 3D bioprinting	Fibroblasts in gelatin/alginate hydrogel	CaCl_2_ solution	Skin wound healing	[82]
	Alginate/polyvinyl alcohol (PVA)	CaCl_2_ solution	As-prepared bone tissue engineering scaffolds	[83]
	Mouse calvaria 3T3-E1 (MC3T3) cells in alginate/PVA/hydroxyapatite (HA) hydrogel	CaCl_2_ solution	Bone tissue engineering	[84]
	Alginate/PVAl/HA/collagen hydrogel	CaCl_2_ solution	Bone tissue engineering	[85]
One nozzle extrusion-based bioploting	Human dental pulp cells (HDPCs) in gelatin/alginate hydrogel	CaCl_2_ solution	Tooth regeneration	[86]
Extrusion-based microvalvebioprinting	Alginate sulfate/nanocellulose/chondrocytes	100 mM CaCl_2_ for 12 min after printing	Cartilage engineering	[87]
One nozzle extrusion-based 3D bioprinting	Human-derived induced pluripotent stem cells (iPSCs) in nanofibrillated cellulose (NFC)/alginate solution	100 mM CaCl_2_ for 5 min prior printing	Cartilage engineering	[88]

**Table 3:** Gelatin containing ‘bioinks’ for different 3D bioprinting applications.

3D Bioprinting Technique	‘Bioink’ Formulation	Crosslinking Method	Application	Ref.
One nozzle extrusion-based 3D low-temperature bioprinting	Hepatocytes in gelatin/chitosan hydrogel	3% sodium tripolyphosphate (TPP)	Hepatic tissue manufacturing	[97]
	Hepatocytes in gelatin hydrogel	2.5% glutaraldehyde	Hepatic tissue manufacturing	[98]
	Hepatocytes in gelatin/fibrinogen hydrogel	Thrombin induced polymerization	Hepatic tissue manufacturing	[99]
	Gelatin/hyluronan	2% glutaraldehyde	Brain tissue repair	[100]
Two-nozzle low-temperature extrusion-based 3D printing	Polyurethane (PU)-gelatin/5% or 10% lysine hydrogel	0.25% glutaraldehyde	Liver manufacturing	[101]
	PU-adipose-derived stem cell (ADSC)/gelatin/alginate/fibrinogen/glycerol or dimethyl sulfoxide (DMSO) hydrogel	Double crosslinking with CaCl_2_ and thrombin solutions	Bioartificial liver manufacturing	[102]
One-syringe extrusion-based 3D printing	Nanosilicate/GelMA	UV light (320–500 nm) for 60 s at an intensity of 6.9 mW/cm^2^	Electrical conductive agent for bone tissue engineering	[103]
EnvisionTEC 3D-Bioplotter^®^	Polyethylene glycol (PEG)/gelatin-PEG/fibrinogen	Gelatin scaffolds were cross-linked with 15 mM EDC and 6 mM NHS, fibrinogen-containing samples were treated post-printing with 10 U/mL thrombin in 40 mM CaCl_2_ for ~30 min	Grid structures for cell seeding	[104]
Dual-syringe Fab@Home printing device	Gelatin ethanolamide methacrylate (GE-MA)-methacrylated hyaluronic acid (HA-MA) (GE-MA-HA-MA)/HepG2 C3A, NIH 3T3, or Int-407 cell	Ultraviolet (UV) light (365nm, 180 mW/cm^2^) photocrosslinking	Tubular hydrogel structures for cell attachment	[105]
Multiple cartridge extrusion-based 3D printer	Polycaprolactone (PCL)-gelatin/fibrinogen/hyaluronic acid/glycerol	Thrombin induced fibrinogen polymerization	Bone, cartilage and skeletal muscle tissues	[91]
One nozzle extrusion-based 3D bioprinting	Human mesenchymal stem cells (MSCs) in gelatin/alginate/hydroxyapatite (HA) mixture	2% *w*/*v* CaCl_2_ crosslinking for 10 min after printing	Bone tissue	[40]
Inkject-based 3D bioprinting	FC3T3 in fibrin-gelatin hydrogel	Thrombin solution	Skin tissue engineering	[106]
